# Performance Evaluation of Non-GPS Based Localization Techniques under Shadowing Effects

**DOI:** 10.3390/s19112633

**Published:** 2019-06-10

**Authors:** Ngoc Mai Nguyen, Le Chung Tran, Farzad Safaei, Son Lam Phung, Peter Vial, Nam Huynh, Anne Cox, Theresa Harada, Johan Barthelemy

**Affiliations:** 1 School of Electrical, Computer and Telecommunications Engineering, University of Wollongong, Wollongong, NSW 2522, Australia; nmn523@uowmail.edu.au (N.M.N.); farzad@uow.edu.au (F.S.); phung@uow.edu.au (S.L.P.); peterv@uow.edu.au (P.V.); 2 Fulbright School of Public Policy and Management, Fulbright University Vietnam, District 7, Ho Chi Minh City 700000, Vietnam; nam.huynh.fsppm@fulbright.edu.vn; 3 School of Management, Operations and Marketing, University of Wollongong, Wollongong, NSW 2522, Australia; avo@uow.edu.au; 4 School of Geography and Sustainable Communities, University of Wollongong, Wollongong, NSW 2522, Australia; tharada@uow.edu.au; 5 SMART Infrastructure Facility, University of Wollongong, Wollongong, NSW 2522, Australia; johan@uow.edu.au

**Keywords:** Non-GPS localization, AOA, RSSI, shadowing effects, VANET

## Abstract

Non-GPS localization has gained much interest from researchers and industries recently because GPS might fail to meet the accuracy requirements in shadowing environments. The two most common range-based non-GPS localization methods, namely Received Signal Strength Indicator (RSSI) and Angle-of-Arrival (AOA), have been intensively mentioned in the literature over the last decade. However, an in-depth analysis of the *weighted* combination methods of AOA and RSSI in shadowing environments is still missing in the state-of-the-art. This paper proposes several weighted combinations of the two RSSI and AOA components in the form of pAOA + qRSSI, devises the mathematical model for analyzing shadowing effects, and evaluates these weighted combination localization methods from both accuracy and precision perspectives. Our simulations show that increasing the number of anchors does not necessarily improve the precision and accuracy, that the AOA component is less susceptible to shadowing than the RSSI one, and that increasing the weight of the AOA component and reducing that of the RSSI component help improve the accuracy and precision at high Signal-to-Noise Ratios (SNRs). This observation suggests that some power control algorithm could be used to increase automatically the transmitted power when the channel experiences large shadowing to maintain a high SNR, thus guaranteeing both accuracy and precision of the weighted combination localization techniques.

## 1. Introduction

Vehicular Ad Hoc Network (VANET) is a network of several moving vehicles and other infrastructures on the road. A car in this network can communicate with other vehicles or Road Side Units (RSUs) to exchange useful information. Positions are the most essential data in VANET since they are used for collision avoidance and congestion prediction. Traditionally, a car can use Global Positioning System (GPS) to locate itself and others. However, GPS does not work effectively in some cases where electromagnetic wave is seriously obstructed by obstacles or even prohibited under some circumstances, such as indoors, underwater [1] and on battle fields. This raises a need of finding an alternative way to locate vehicles without using GPS.

Non-GPS localization is categorized into two streams, namely range-free and range-based methods [2]. Range-based methods take advantage of additional hardware on the car to estimate distances or angles [3,4,5] among the nodes. These estimations will then be utilized to find the position of the node with respect to the anchors (landmark nodes). On the other hand, range-free methods use signal coverage to define a region that definitely contains an unknown node [6,7]. The region can be refined further using several complex algorithms. The unknown node is then estimated to be centroid [8,9] or chosen randomly inside this region [10]. Because range-free methods do not have any information about the network but the signal coverage, they need a denser network than the range-based methods in order to locate nodes. Although the range-free methods are cheaper because of their simpler hardware requirements, their accuracy is generally lower than that of the range-based ones. The range-based methods generally require additional hardware to help nodes obtain prior knowledge of the network, including bearing measurements such as angles or distances between vehicles. Based on this information, a vehicle can find its position more accurately. Therefore, the range-based methods normally produce a better performance than the range-free ones, especially in a noisy environment [11].

As a result, this paper focuses on the range-based methods. In particular, we consider the two most popular representatives in the range-based methods. The first method is Received Signal Strength Indicator (RSSI) [6,7,8,9,10,11,12,13,14,15], which is well known for its hardware simplicity but subjects to errors in noisy and shadowing environments. The second method is Angle-of-Arrival (AOA) [3,4,5,16], which requires adoption of an antenna array in the vehicles or at the anchors but is greatly resilient to noise. Extensive work has been carried out to evaluate RSSI and AOA performances individually in the literature over the last ten years [14,17,18,19,20,21,22,23,24,25,26,27,28,29,30]. However, their combination has not been thoroughly analyzed, especially in the shadowing environments.

Therefore, this paper proposes and evaluates various combinations with different weights of the two RSSI and AOA components in terms of accuracy and precision. The contributions of this paper include:

- review of the state-of-the art non-GPS localization techniques with the focus on RSSI and AOA methods;
- proposal of combined RSSI-AOA localization methods with different weights of the components RSSI and AOA;
- comprehensive summary on working concepts of these combined methods;
- introduction of the localization model under shadowing effects; and
- numerous simulations and in-depth discussions on the precision and accuracy of the proposed combined localization techniques under shadowing effects.

The paper is outlined as follows. Section 2 reviews some related works. Section 3 proposes the combined RSSI-AOA methods with different weights for the RSSI and AOA components. Section 4 derives the localization model taking into account the shadowing effects. Section 5 presents simulation results and analyses of their performances under shadowing effects. Section 6 concludes the paper and presents future work.

*Notations*: Throughout this paper, the following notations are used. $\sigma^{2}$ represents the noise variance affecting received antennas. $\delta^{2}$ denotes the variance of shadow. For brevity, we denote 1AOA + 1RSSI for the combined AOA-RSSI localization method where the unknown node is positioned by *one* distance between the unknown node and an anchor determined based on the RSSI method and *one* incident angle from the unknown node to the antenna array at this anchor node determined based on the AOA method. The notation 1AOA + 2RSSI denotes the combined method where *one* incident angle and *two* distances to the two anchor nodes are used to position the unknown node. Similarly, we use the following notations: 2AOA + 1RSSI, 2AOA, 2RSSI, 2AOA + 2RSSI, and 3RSSI. We refer to these localization methods to as the *weighted combinations* of AOA and RSSI.

## 2. Related Works

Many methods have been proposed in the range-based category, including RSSI, AOA, Time Difference-of-Arrival (TDOA), Power Difference-of-Arrival (PDOA), and Frequency Difference-of-Arrival (FDOA). Each method has its own strengths and weaknesses and there is not yet a dominant technique to solve all problems. These methods are summarized in detail in Table 1.

Numerous researchers have recently tried to overcome the disadvantages of individual approaches by joining different approaches. A common combination is to use AOA with other methods. Typical combinations are AOA with TOA [31], and AOA with TDOA [32]. Simulations in [31,32] have shown that such combinations can produce a better accuracy in ideal situations. However, the methods AOA + TOA and AOA + TDOA require a perfect synchronization. The most popular combination is between AOA and RSSI methods since it does not require a perfect synchronization. For example, Dai et al. [33] proposed to use RSSI to locate nodes and then use AOA to refine the result. This makes the computation process longer and complicated. It also requires at least three anchor nodes, which is not always feasible in VANETs. On the other hand, the localization models in [30,34,35] do not consider shadowing effects. The authors of [24,25] used the same approach of using RSSI to compute AOA by rotating a directional antenna at a pre-defined angle and measuring the received power at that angle. The receiver can then gain the power spectrum of all possible arrival angles and find the arrival angle which is corresponding to the highest received power in the spectrum. Due to the time delay required to estimate the arrival angle as a result of antenna rotation, this method seems to be infeasible in practice.

Given that shadowing effects are indispensable in VANETs, especially in urban and suburban areas, this paper concentrates on the analysis of weighted combinations of AOA and RSSI under shadowing and noise effects.

### 2.1. Distance and Angle Estimation Concepts

#### 2.1.1. RSSI

RSSI takes advantage of the fact that the distance is, in an ideal scenario, inversely proportional to the received signal strength. However, shadowing will significantly affect this relation. For illustration, we consider the path loss model in [2] to compute RSSI as below

(1) $$\begin{array}{ccc} RSSI & = & P_{T}-PL\left(d\right)\\ PL(d) & = & PL\left(d_{0}\right)+10n\log_{10}(\frac{d}{d_{0}})+W, \end{array}$$

where $P_{T}$ (dB) is the transmitted power, PL(d) (dB) is the path loss at distance *d*, $PL(d_{0})$ (dB) is the path loss of at the unit distance $d_{0}$, *n* is the path loss exponent, and $W∼N(0,\delta^{2})$ (dB) is a Gaussian random variable representing the shadowing effects with zero mean and the variance $\delta^{2}$.

By rearranging Equation (1), the distance from the unknown node to the anchor node can be calculated as

(2) $$d=d_{0}\times 10^{\frac{PL\left(d\right)-PL\left(d_{0}\right)-X}{10n}}.$$

#### 2.1.2. AOA

To detect the AOA of an incoming signal, a uniform antenna array is usually used at the receiver. Among many proposed methods to calculate AOA, Multiple Signal Classification (MUSIC) is the most dominant and simplest method [69]. Hence, we choose the MUSIC algorithm to estimate the AOA. The working concept of MUSIC is as follows.

Assuming that there are *K* incoming signals, each of which is observed by a limited number $N_{S}$ of snapshots. Denote X as the $K\times N_{S}$ matrix of transmitted signals and N as the $M\times N_{S}$ additive noise matrix, whose noise samples follow $\mathrm{CN}(0,\sigma^{2})$. The received signal matrix at the receive antenna array will be

(3) Y=A(θ)X+N,

where $A\left(\theta \right)=[a\left(\theta_{1}\right),\ldots ,a\left(\theta_{K}\right)]$ is a steering matrix, and $\theta_{k}$ and $a(\theta_{k})$ are the angle of arrival and the steering vector for the *k*th incoming signals (k=1,…,K). The steering vector of an antenna array is $a\left(\theta_{k}\right)={\left[1\;e^{j(kd_{a}sin\left(\theta_{k}\right))}\ldots e^{j(\left(M-1\right)kd_{a}sin\left(\theta_{k}\right))}\right]}^{T}$ where *M* is the number of antenna elements in each antenna array, $k=\frac{2\pi }{\lambda }$ is the wave number, λ is the wavelength, and $d_{a}$ is the distance between two adjacent antenna elements.

MUSIC algorithm estimates the AOA by correlating the received signal. Covariance matrix $R_{yy}$ of the received signal matrix Y is calculated as

(4) $$R_{yy}=AR_{xx}A^{H}+\sigma^{2}R_{nn},$$

where $R_{xx}$ is the covariance matrix of the transmitted signal matrix X, and $R_{nn}$ is the covariance matrix of noises normalized by $\sigma^{2}$, i.e., $R_{nn}=E\left\{NN^{H}\right\}/\sigma^{2}$. In the ideal case where the noise samples are all independent, $R_{nn}$ is an identity matrix I. In reality, $R_{nn}$ is not an identity one, thus affecting significantly the performance of MUSIC. Both ideal and realistic cases will be simulated in Section 5 of this paper.

Eigenvalue decomposition is then applied to $R_{yy}$. It is well known that eigenvalues represent the power of incoming signals. Thus, if the eigenvalues are rearranged in an ascending order, the last *K* eigenvalues correspond to the *K* incoming signals, while the M−K remaining eigenvalues correspond to noises. Denote $U=[u_{1},\ldots ,u_{M-K}]$ as the matrix of eigenvectors, which correspond to the eigenvalues of noises, then U represents the noise space.

MUSIC method is a search-based algorithm which assumes that the received signals are independent to noise. As a result, in theory, the noise space U and the steering vector a(θ) are orthogonal, i.e., the Euclidean distance $a{\left(\theta \right)}^{H}U^{H}Ua\left(\theta \right)=0$, at each and every angle of arrival $\theta_{1},\ldots ,\theta_{K}$, where ${\left(.\right)}^{H}$ denotes the Hermitian transpose matrix. In reality, the noise space and steering vectors cannot be perfectly orthogonal to each other due to errors. However, their multiplication is still relatively small. MUSIC takes advantage of this feature to estimate the incoming angles by searching for the angles $\theta^{\prime }$ which produce the peak of the below power spectrum

(5) $$S_{MUSIC}\left(\theta^{\prime }\right)=\frac{1}{|a{\left(\theta^{\prime }\right)}^{H}U^{H}Ua\left(\theta^{\prime }\right)|},$$

where $S_{MUSIC}\left(\theta^{\prime }\right)$ is the power spectrum of $\theta^{\prime }$ and $\theta^{\prime }\in \left[-\pi /2,\pi /2\right]$.

As mentioned above, in the ideal case, noise samples are assumed to be independent, thus $R_{nn}=I$. The noise subspace is easy to be separated from the transmitted signal subspace, thus MUSIC has a very high accuracy. However, in practice, noise samples at the receiver are not totally independent. The noise subspace is harder to be separated accurately from the transmitted signal subspace. As a result, the performance of MUSIC experiences some degree of deterioration. More details about this deterioration are given in Section 5.

### 2.2. Existing Localization Methods

In this section, we discuss mathematical formulas and algorithms using angle and distance measurements obtained from Section 2.1 to position an unknown node. All of these algorithms were simulated and compared with our weighted combination algorithms, as presented in Section 5.

**Figure 1:**
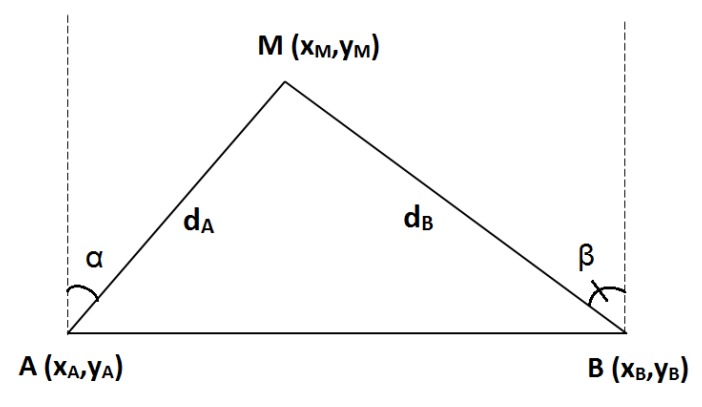
Localization using 2AOA.

Assuming that the network contains up to three anchor nodes, denoted as A, B and C, the unknown node whose position is in question is denoted as M (cf. Figure 1). We assume that the unknown node periodically sends out a Radio Frequency (RF) signal asking for its updated position. The anchors will then calculate and send back the unknown node’s position based on the received signal. Denote the distances between M and the anchors as $d_{A}$, $d_{B}$ and $d_{C}$, and the angles of arrival at the antenna arrays at anchors A and B as α and β (α,β∈[−π/2,π/2]), respectively. Clearly, distances can be estimated by the RSSI method while AOA can be estimated by the MUSIC algorithm. Some well-known localization methods in the literature are briefly reviewed as follows.

#### 2.2.1. Triangulation (2AOA)

In this method, the incident angles α and β at the two anchors A and B (cf. Figure 1) are first estimated by MUSIC. The unknown node M is located as the intersection between two angular directions without any RSSI measurement. Since this method relies on two angular measurements, it is denoted as 2AOA. Coordinates of M are worked out as below.

(6) $$\begin{array}{ccc} y_{M} & = & y_{A}+\frac{x_{B}-x_{A}}{\tan\left(\alpha \right)-\tan\left(\beta \right)}\\ x_{M} & = & x_{A}+\left(y_{M}-y_{A}\right)\tan\left(\alpha \right). \end{array}$$

The above equations can be easily proved by replacing $\tan\left(\alpha \right)=\frac{x_{M}-x_{A}}{y_{M}-y_{A}}$ and $\tan\left(\beta \right)=\frac{x_{M}-x_{B}}{y_{M}-y_{B}}$.

In Equation (6), without loss of generality, we assume that A and B are placed in a line parallel to the horizontal axis of the Cartesian coordinate, thus $y_{A}=y_{B}$. This assumption is still valid because non-GPS localization does not calculate the absolute coordinates of M. Instead, the relative coordinates of M with respect to those of the anchor nodes are sufficient.

#### 2.2.2. Trilateration with Two Anchors (2RSSI)

**Figure 2:**
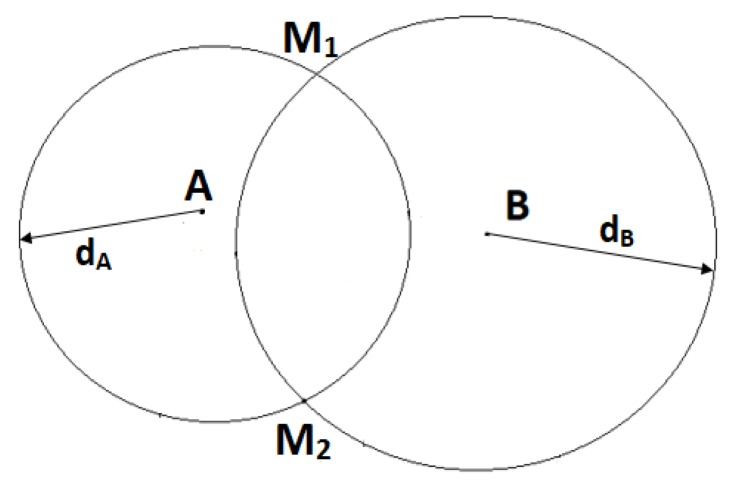
Localization using 2RSSI.

This method, referred to as 2RSSI, uses two estimated distances $d_{A}$ and $d_{B}$, which can be found by the RSSI method, to find the unknown node. As shown in Figure 2, there are two circles centered at A and B with the radii of $d_{A}$ and $d_{B}$. Theoretically, M can be at one of the two intersections between these two circles ($M_{1}$ or $M_{2}$). In practice, a car may only need to locate other vehicles in front of it. Therefore, it is reasonable to assume that the unknown node is in the upper plane above the line AB. As a result, in Figure 2, $M_{1}$ will be chosen to be the position of the unknown node M.

#### 2.2.3. Trilateration with Three Anchors (3RSSI)

**Figure 3:**
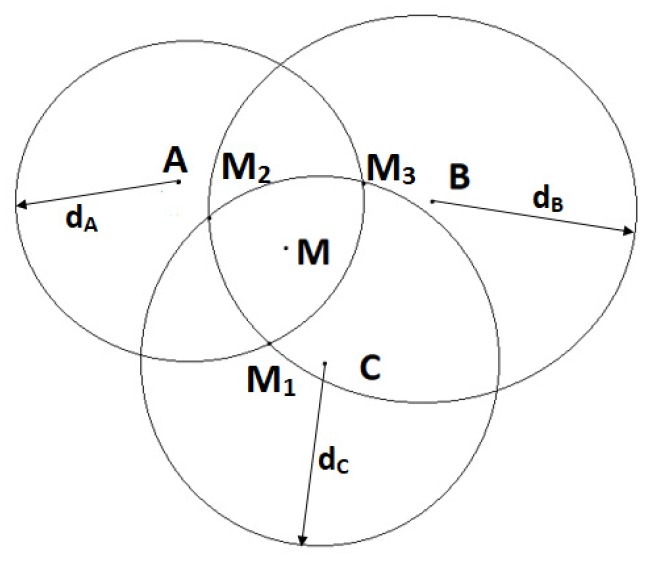
Localization using 3RSSI.

The distances $d_{A}$, $d_{B}$ and $d_{C}$ from the unknown node to the three anchors are estimated using the RSSI method. As explained in Section 2.2.2, each distance pair can produce two intersection points. Because there are three estimated distances, $d_{A}$, $d_{B}$ and $d_{C}$, there will be six intersection points, as shown in Figure 3. The three most close intersection points to each others are the ones of our interest. The coordinates of the unknown node M will be simply the average of the coordinates of these three points.

#### 2.2.4. Weighted Centroid Method (weighted 3RSSI)

Because the received power is inversely proportional to the distance, RSSI measurements will be more erroneous when the unknown node moves further from the anchors. To reduce the effect of RSSI measurement errors, Shi et al. [62] proposed the weighted centroid algorithm, which places a higher weight on the RSSI measurement for a closer anchor node. This algorithm follows Equation (7) as below

(7) $$\begin{array}{ccc} x_{M} & = & \frac{\frac{x_{1}}{d_{A}+d_{B}}+\frac{x_{2}}{d_{B}+d_{C}}+\frac{x_{3}}{d_{A}+d_{C}}}{\frac{1}{d_{A}+d_{B}}+\frac{1}{d_{B}+d_{C}}+\frac{1}{d_{A}+d_{C}}}\\ y_{M} & = & \frac{\frac{y_{1}}{d_{A}+d_{B}}+\frac{y_{2}}{d_{B}+d_{C}}+\frac{y_{3}}{d_{A}+d_{C}}}{\frac{1}{d_{A}+d_{B}}+\frac{1}{d_{B}+d_{C}}+\frac{1}{d_{A}+d_{C}}}, \end{array}$$

where $\left(x_{1},y_{1}\right),\;\left(x_{2},y_{2}\right)$ and $\left(x_{3},y_{3}\right)$ are coordinates of three intersections $M_{1}$, $M_{2}$ and $M_{3}$, respectively (cf. Figure 3); and $d_{A},\;d_{B},\;d_{C}$ are distances from A, B and C to M, measured based on the RSSI method.

Equation (7) assigns different weighting factors to $M_{1},M_{2},M_{3}$. The weights are chosen to compensate the estimation error due to power attenuation. For instance, as the unknown node *M* moves further away from anchors A and B, $d_{A}$ and $d_{B}$ increase and thus might be estimated inaccurately. The corresponding weighting factor decreases accordingly and, therefore, the final estimated unknown node will depend less on the intersection $M_{1}=\left(A,d_{A}\right)\cap \left(B,d_{B}\right)$. The same principle is applied to $M_{2}$ and $M_{3}$.

## 3. Localization Using Combinations of RSSI and AOA

Traditionally, a node is usually found by distance measurements (i.e., trilateration), angle measurements (triangulation), or a simple combination of the two methods. However, in this paper, we propose the combinations of RSSI and AOA with different weights of these components. In this section, we explain in detail how an unknown node is positioned using various mixtures of distance and angle measurements. For brevity, we use the notations of the combined AOA-RSSI methods as explained at the end of Section 1.

### 3.1. 1AOA + 1RSSI

This method is the simplest combination of the two methods. The angle measurement and the distance measurement are equally important in determining the position of M. It converts polar coordinates into Cartesian coordinates to locate the node M with respect to the anchor node A as below

(8) $$\begin{array}{ccc} x_{M} & = & x_{A}+d_{A}\sin\left(\alpha \right)\\ y_{M} & = & y_{A}+d_{A}\cos\left(\alpha \right), \end{array}$$

where α is the AOA of the signal which is transmitted from Node M and arrives at Node A.

The advantage of this method is that we can still locate nodes with a minimum of one anchor node. The performance under this extreme case is analyzed further in Section 5.

### 3.2. 1AOA + 2RSSI

In contrast to the previous technique, this technique uses two anchors. It puts more weight on the RSSI measurements than the AOA ones, using three parameters $\left(\alpha ,d_{A},d_{B}\right)$. First, the 2RSSI technique (see Section 2.2.2) is used, giving an estimated position $M_{1}$ of the node M. Next, the 1AOA + 1RSSI technique mentioned in Section 3.1 provides another estimated position $M_{2}$ of the node M. The position of M will then be calculated as the average of the two located nodes $M_{1}$ and $M_{2}$.

### 3.3. 2AOA + 1RSSI

Similar to 1AOA + 2RSSI, two anchors are used in this method. However, it puts more weight on the AOA component. Three parameters $\left(\alpha ,\beta ,d_{A}\right)$ are used to locate M following the below equations

(9) $$\begin{array}{ccc} y_{M} & = & y_{A}+\frac{1}{2}[d_{A}\cos\left(\alpha \right)+\frac{x_{B}-x_{A}}{\tan\left(\alpha \right)-\tan\left(\beta \right)}]\\ x_{M} & = & x_{A}+\frac{1}{2}[d_{A}\sin\left(\alpha \right)+\left(y_{M}-y_{A}\right)\tan\left(\alpha \right)] \end{array}$$

### 3.4. 2AOA + 2RSSI

Two anchors and four parameters $\left(\alpha ,\beta ,d_{A},d_{B}\right)$ are used in this technique. In particular, an estimated position $M_{1}$ of the unknown node M is worked out from two parameters $\left(\alpha ,d_{A}\right)$ by the above 1AOA + 1RSSI method (cf. Equation (8))

(10) $$\begin{array}{ccc} x_{M_{1}} & = & x_{A}+d_{A}\sin\left(\alpha \right)\\ y_{M_{1}} & = & y_{A}+d_{A}\cos\left(\alpha \right). \end{array}$$

Further, from two parameters $\left(\beta ,d_{B}\right)$, another estimated position $M_{2}$ can be found by the 1AOA + 1RSSI technique, i.e.,

(11) $$\begin{array}{ccc} x_{M_{2}} & = & x_{B}+d_{B}\sin\left(\beta \right)\\ y_{M_{2}} & = & y_{B}+d_{B}\cos\left(\beta \right). \end{array}$$

In an ideal condition without noise and shadowing, the positions of $M_{1}$ and $M_{2}$ will be coincident. However, due to noise and shadowing effects, $M_{1}$ and $M_{2}$ are found at different locations. The position of M is chosen to be the middle point of these two estimated points. Therefore, coordinates of M are calculated as

(12) $$\begin{array}{ccc} x_{M} & = & \frac{1}{2}[x_{A}+x_{B}+d_{A}\sin\left(\alpha \right)+d_{B}\sin\left(\beta \right)]\\ y_{M} & = & \frac{1}{2}[y_{A}+y_{B}+d_{A}\cos\left(\alpha \right)+d_{B}\cos\left(\beta \right)]. \end{array}$$

Table 2 summarizes all aforementioned localization methods with the detailed number of required anchors, required parameters, mathematical formulas, and the corresponding graphical illustrations for the ease of understanding.

## 4. Localization under Shadowing Effects

Equations (8)–(12) involve the calculation of distances, such as $d_{A}$ and $d_{B}$. As shown in Equation (1), shadowing effects have a significant impact on the accuracy of distance calculations. Equations (8)–(12) also involve the calculation of angles of arrival based on the eigenvalue decomposition of the covariance matrix $R_{yy}$, as shown in Equation (4). Under the effect of shadowing, the covariance matrix $R_{yy}$ will change, as do its eigenvalues.

To compare fairly all the weighted combination methods mentioned in Section 3 under shadowing effects, we consider the following communication model between any two nodes. Assuming x(t) is the transmitted RF signal at the time instant *t*, we have

(13) $$x\left(t\right)=ℜ\left\{\sqrt{P_{T}}\;se^{j2\pi f_{c}t}\right\},$$

where ℜ{.} denotes the real part; *s* is the baseband signal with unit average power, i.e., $E\{|s{|}^{2}\}=1$; $P_{T}$ is the transmitted power; and $f_{c}$ is the carrier frequency. Define the Signal-to-Noise Ratio SNR as $SNR=\frac{P_{T}}{\sigma^{2}}$, where $\sigma^{2}$ denotes the noise power.

The received signal y(t) has the power $P_{R}$ calculated in dB as $P_{R}=P_{T}-PL\left(d\right)$, where $PL\left(d\right)=PL\left(d_{0}\right)+10\log_{10}\left(\frac{d}{d_{0}}\right)+W$ (cf. Equation (1)) represents the path loss (dB) at the distance *d*. *W* represents the shadowing effect, which is modeled as a Gaussian random variable with zero mean and variance $\delta^{2}$. The received RF signal in a flat fading channel is modeled as

(14) $$\begin{array}{ccc} y(t) & = & ℜ\{\sqrt{P_{R}}\;sh\;e^{j2\pi f_{c}\left(t-\tau \right)}+n\left(t\right)\}, \end{array}$$

where $\tau =\frac{d}{c}$ is the propagation delay; *d* is the distance between the transmitter and the receiver; *c* is the speed of light; *h* represents the complex channel coefficient, i.e., the small scale fading, and is modeled as a complex Gaussian random variable following CN(0,1); and n(t) is random noise which is assumed to follow the distribution $\mathrm{CN}(0,\sigma^{2})$.

The model in Equation (14) includes both small scale fading and shadowing effects. Under the shadowing effect, the average received power varies, thus affecting the precision and accuracy of the RSSI and AOA estimations, as detailed in Section 5 below.

## 5. Simulation Results and Analyses

### 5.1. Precision

We ran simulations in MATLAB™ to evaluate the precision of various weighted combination methods with and without shadowing effects. We assumed that unknown nodes have random positions within a 100 m × 100 m rectangle. $P_{T}$ and $f_{c}$ were initialized to be 1 W and 100 kHz, respectively. The unit distance and path loss exponent were set as 1 m and 2 m, respectively.

**Figure 4:**
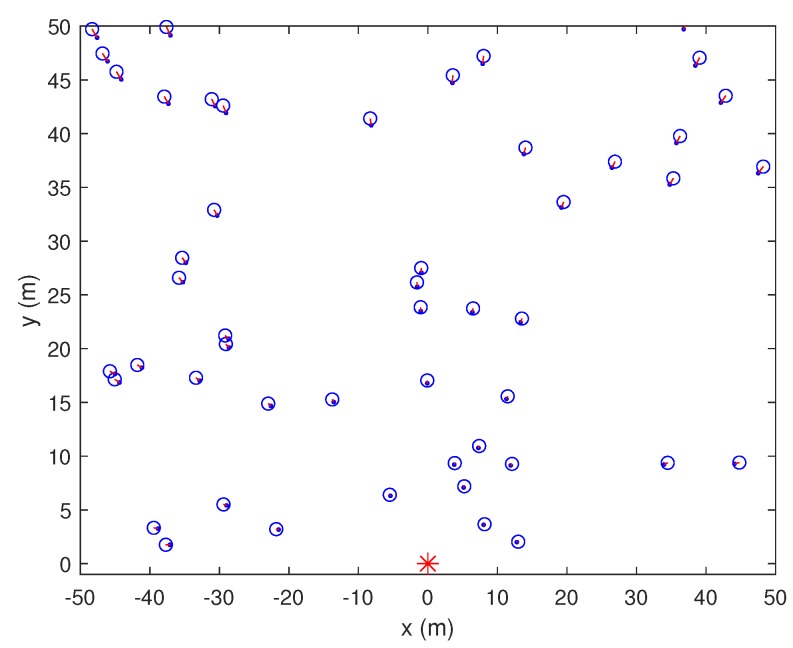
1AOA + 1RSSI method without shadowing effects ($\delta^{2}=0$).

**Figure 5:**
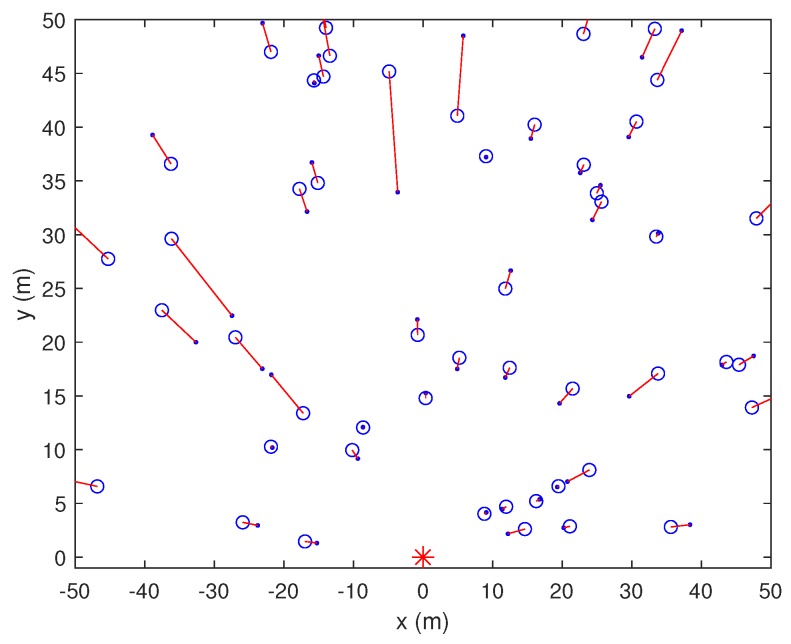
1AOA + 1RSSI method under shadowing effects ($\delta^{2}=1$).

**Figure 6:**
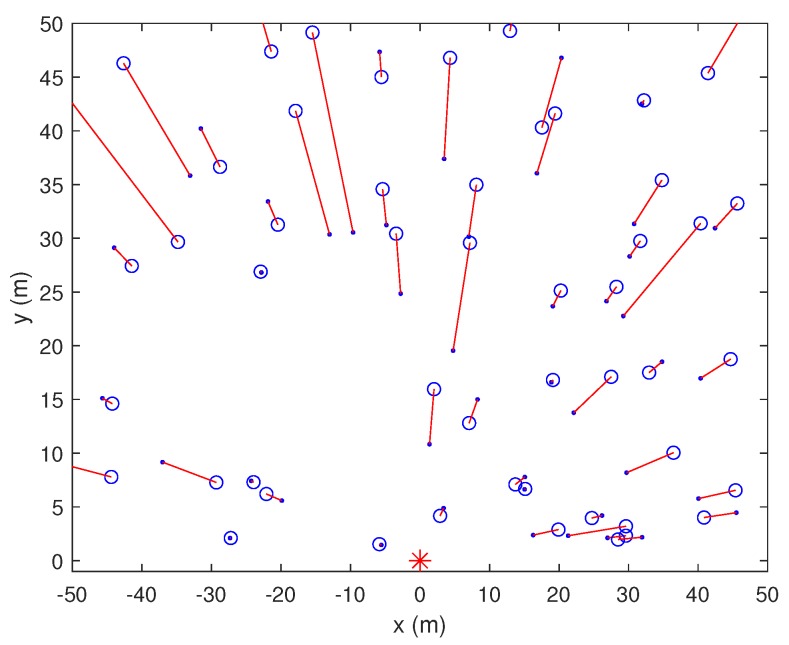
1AOA + 1RSSI method under shadowing effects ($\delta^{2}=4$).

We first compared the shadowing effects in localization using one anchor node. Figure 4, Figure 5 and Figure 6 show the results for 1AOA + 1RSSI with and without shadowing effects at SNR = 15 dB. The blue circle is the located node, the blue dot is the original position, and the asterisks denote the anchors. The anchor at the coordinate origin, referred to as Node A, is of our interest in Figure 4, Figure 5 and Figure 6. From these figures, the following observations can be drawn. When shadowing effects are negligible, e.g., in the normal countryside terrain, both angle and distance estimations of 1AOA + 1RSSI are relatively accurate. When shadowing effects increase to $\delta^{2}=1$ and $\delta^{2}=4$, the accuracy of the angle and distance estimations deteriorates, thus the location errors increase. It can be seen from Figure 5 and Figure 6 that, when $\delta^{2}$ increases from 1 to 4, most of the red lines connecting the true positions and the estimated positions of the unknown nodes still orient towards the anchor located at the coordinate origin. Meanwhile, the distance errors become noticeably larger. This means that, compared to the angle estimations, distance estimations are more susceptible to the shadowing effects. In addition, the errors (mainly distance errors) increase when the unknown node is further away from the anchor. This occurs because the signal power attenuates more when the distance between the receiver and the transmitter increases, which makes distance estimations become inaccurate. Consequently, the unknown node might be wrongly located.

Next, we compared the weighted combinations of AOA and RSSI mentioned in Section 2.2 in terms of precision. In our simulations, 200,000 unknown nodes were placed randomly within the rectangle. To compare the effects of noise and shadowing, all remaining graphs in this section plot the average Mean Square Error (MSE) of the distance between the true position and the estimated one of the unknown nodes in each method with respect to SNR. The MSE at a SNR is defined as follows

(15) $$MSE=\frac{1}{N}\sum_{i=1}^{N}{\left(\frac{\Delta R_{i}}{R_{i}}\right)}^{2}$$

where $R_{i}$ denotes distance from the estimated node to the first anchor (Node A), $\Delta R_{i}$ is the distance from the actual position of the unknown node to its estimated position, and *N* is the number of unknown nodes which can be successfully located (perhaps with errors) for this particular SNR. Sometimes *N* can be smaller than 200,000 because the node cannot be computed due to noise and shadowing.

#### 5.1.1. Localization with Ideal Covariance Matrix of Noise

**Figure 7:**
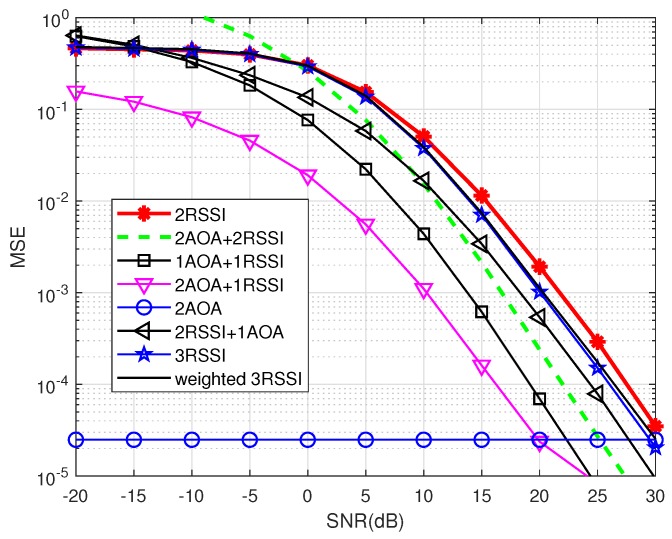
Precision comparison using ideal covariance noise matrix without shadowing effects ($\delta^{2}=0$).

**Figure 8:**
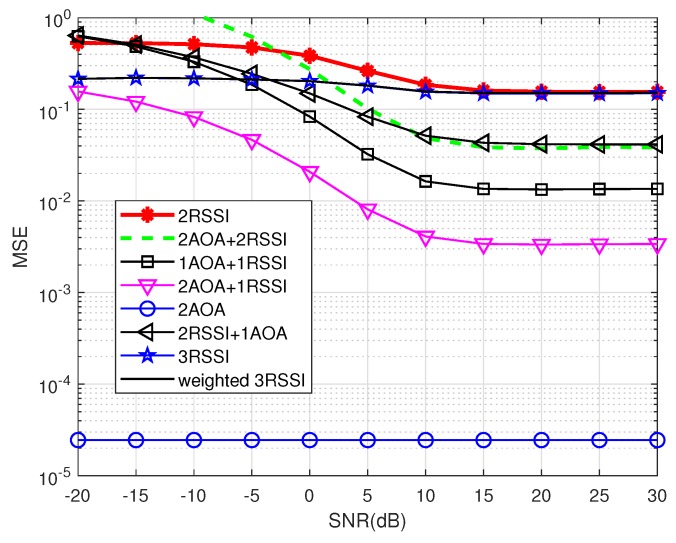
Precision comparison using ideal covariance noise matrix under shadowing effects ($\delta^{2}=1$).

**Figure 9:**
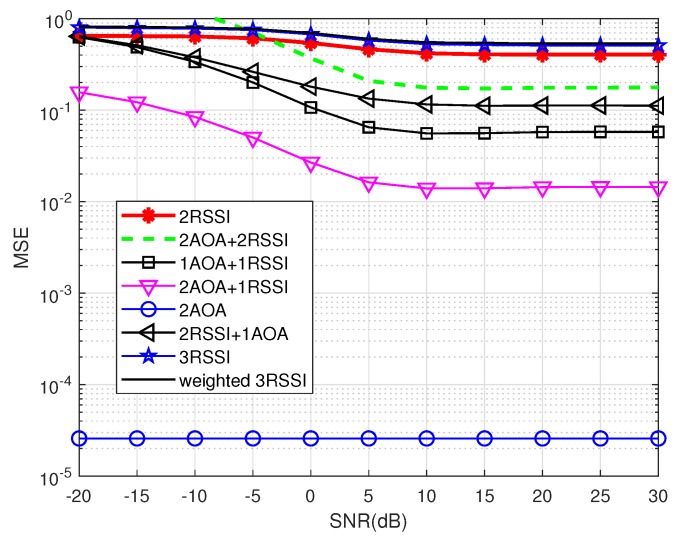
Precision comparison using ideal covariance noise matrix under shadowing effects ($\delta^{2}=4$).

In the ideal case, the covariance noise matrix is an identity matrix, as mentioned in Section 2.1.2. Figure 7, Figure 8 and Figure 9 show the localization precision under increasing shadowing effects with $\delta^{2}=0$, 1 and 4, respectively.

In the low-to-medium SNR range, the 2AOA method has the best precision with MSE being constant at $10^{-4.9}$ when SNR varies. This comes from the fact that, in the ideal case, all off-diagonal entries of the noise covariance matrix are assumed to be zeros, thus MUSIC is likely able to separate accurately the signal subspace and the noise subspace, regardless of shadowing effects. Unlike the 2AOA, all remaining methods have the component RSSI, thus their precision is significantly affected by noise and shadowing effects. (When SNR increases, the performance of RSSI improves because the estimated distance converges to the true value.) This proves that AOA is more resilient not only to noise but also to shadowing effects, compared to RSSI.

This less susceptibility of the AOA component to noise and shadowing, compared to the RSSI component, explains the fact that the localization methods using bigger weights for the AOA component than for the RSSI component *more likely* outperform the remaining methods. For example, the precision of 2AOA and 2AOA + 1RSSI are much better than that of 2RSSI and 1AOA + 2RSSI. However, some exceptions exist as mentioned at the end of this subsection.

This more resilience to noise and shadowing also explains for the observation that the combination approaches, i.e., 1AOA + 2RSSI, 1AOA + 1RSSI and 2AOA + 1RSSI, are always better, especially at high SNRs, than the pure RSSI methods, namely 2RSSI, 3RSSI and weighted 3RSSI. In other words, increasing the number of anchors to three in the 3RSSI and weighted 3RSSI methods does not improve the precision as much as the combination approaches do, even with only two anchor nodes.

From these figures, it is clear that, except the 2AOA method, the precision in all other methods degrades significantly when shadowing effects increase. It can also be observed that 2AOA + 2RSSI performs worse than 1AOA + 1RSSI, which is an exception case, as mentioned before, because of the error of 2AOA + 2RSSI in averaging two located nodes, and that the overall performance of the weighted 3RSSI is almost the same as that of the 3RSSI, especially when shadowing presents, because the error in distance estimations outweighs the advantage of using weighting factors in the weighted 3RSSI method. These observations indicate that increasing the number of anchor nodes does not automatically guarantee a better performance.

#### 5.1.2. Localization with Correlated Noises

The covariance matrix of noise is ideally an identity matrix. This occurs when many realizations (samples) of noise are considered over time and these realizations are assumed to be independent of each other. However, in realistic applications, the anchor nodes have to predict the location of the unknown node (e.g., a moving car) in real time. Thus they are only able to observe a limited number of time samples of the noisy signal Y (cf. Equation (3)) received from the moving node before estimating the angle-of-arrival of this node. In other words, at any given time instant, the anchor nodes can only perform the eigenvalue decomposition of the instantaneous value of $YY^{H}$ to detect the angle-of-arrival immediately, rather than the eigenvalue decomposition of its expectation value $E\{YY^{H}\}$. That is, the instantaneous value of $NN^{H}$ is involved, instead of $R_{nn}=E\left\{NN^{H}\right\}$ as in the theoretical MUSIC algorithm (cf. Equation (4)). Clearly, $NN^{H}$ is not an identity matrix, i.e., there exist off-diagonal elements. These off-diagonal elements affect the eigenvalue decomposition of $YY^{H}$, which might cause the faulty separation between the signal space and the noise space, i.e., the localization errors.

**Figure 10:**
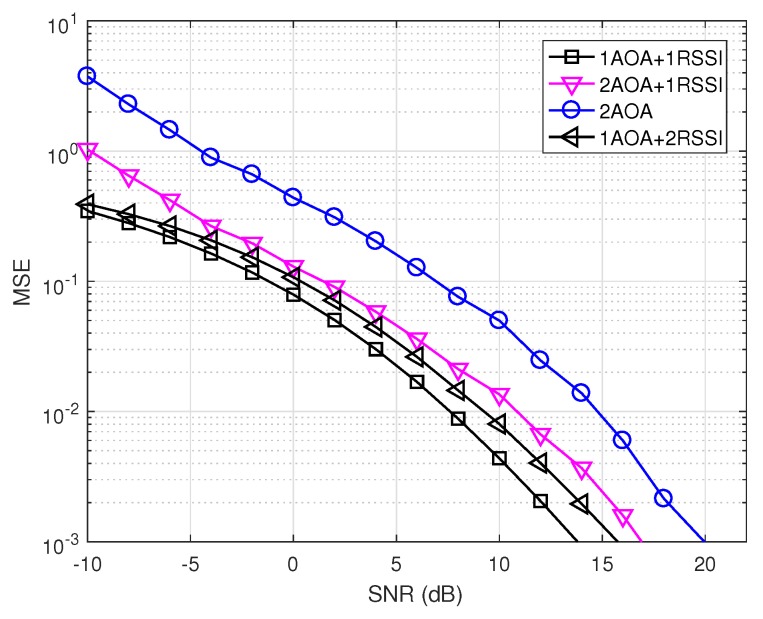
Precision comparison using instantaneous realizations of noise without shadowing ($\delta^{2}=0$).

**Figure 11:**
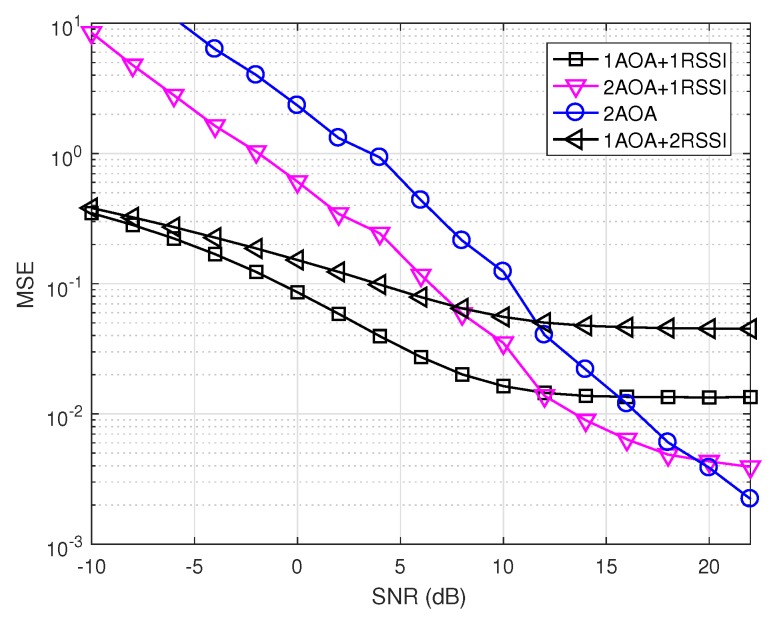
Precision comparison using instantaneous realizations of noise with shadowing ($\delta^{2}=1$).

We generated instantaneous realizations of noise in MUSIC as mentioned in Section 2.1.2. Figure 10 and Figure 11 show the precision performance of four methods without and with shadowing effects. Unlike the ideal case where the AOA method performs best, it has the worst performance among the four compared methods at the lower SNR range in both Figure 10 and Figure 11. This shows that the noise correlation degrades significantly the accuracy of the angle estimations in MUSIC, unless SNR is large enough.

Figure 11 shows that the localization precision of all methods deteriorates under shadowing effects. In addition, all methods having the RSSI component experience the saturation phase when SNR increases, similarly to the ideal case (cf. Figure 8 and Figure 9). This means shadowing effects have a significant impact on RSSI-based methods. It can also be observed that 2AOA and 2AOA + 1RSSI outperform other RSSI related methods at high SNRs. Similar to the ideal case, these observations confirm that the AOA component is affected more by noise than by shadowing effects, compared to the RSSI component. The AOA component has low precision at low SNRs, but its performance quickly improves when SNR increases. As a result, the 1AOA + 1RSSI method performs best in the low SNR range, the 2AOA + 1RSSI performs the best in the medium SNR range, and, in the high SNR range, the 2AOA method performs best.

This result is promising since 2AOA and 2AOA + 1RSSI outperform other RSSI related methods at medium-to-high SNRs in a shadowing environment. This observation indicates that some adaptive power control algorithm can be used to improve significantly the precision of these two methods in the shadowing environment. The adaptive power control will automatically increase the transmitted signal power if the propagation channel experiences shadowing effects, thus maintaining the high SNR value. Therefore, the two methods 2AOA and 2AOA + 1RSSI, assisted by an adaptive power control algorithm, will be two potential candidates for an environment possessing strong shadowing, such as urban areas.

From Figure 7, Figure 8, Figure 10 and Figure 11, there is a huge difference in the performance of 2AOA using the ideal covariance matrix and the instantaneous noise realizations. While 2AOA performs perfectly in the former, it performs poorly at low SNRs in the latter. Another observation is the AOA-related methods using instantaneous noise realizations have a tendency to level off at high SNRs and approach those using the ideal matrix.

**Figure 12:**
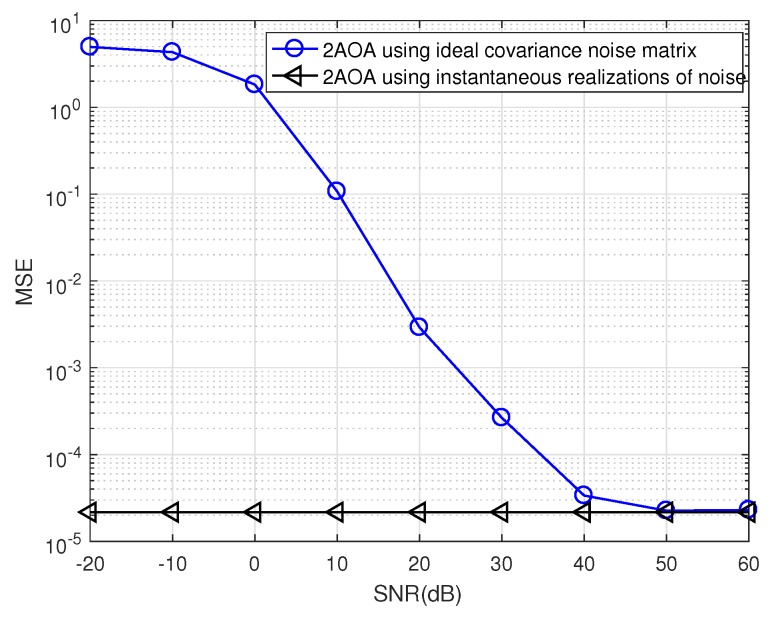
Confirmation of the lower bound of the AOA performance ($\delta^{2}=0$).

To confirm this, Figure 12 plots the performance of 2AOA in the two cases. As predicted, two lines converge when SNR is really high. There is a big gap between these two lines when SNR is low. The reason is that, at a low SNR, the off-diagonal entries in the instantaneous noise covariance matrix are of significant values, making MUSIC fail to separate between the signal subspace and the noise subspace from the eigenvalues of the matrix $R_{yy}$ (cf. Equation (4)). In the ideal case, all off-diagonal entries are zeros, thus MUSIC is able to separate the two subspaces. Figure 12 proves that the curve in the ideal case is the lower bound of the realistic curve. Therefore, the ideal noise model might be useful in predicting the realistic performance at a high SNR. This simplifies the computation complexity of the algorithm.

### 5.2. Accuracy

We compared performance in terms of accuracy, which was defined as the Relative Distance Error (RDE) calculated as follows:

(16) $$RDE=\frac{\Delta R}{R_{0}}\times 100$$

where ΔR is the distance between the original and the located unknown node, $R_{0}$ is the distance from the real position of the unknown node to the first anchor. RDE represents how far a node is located from its exact position relatively to its distance to the first anchor node.

**Figure 13:**
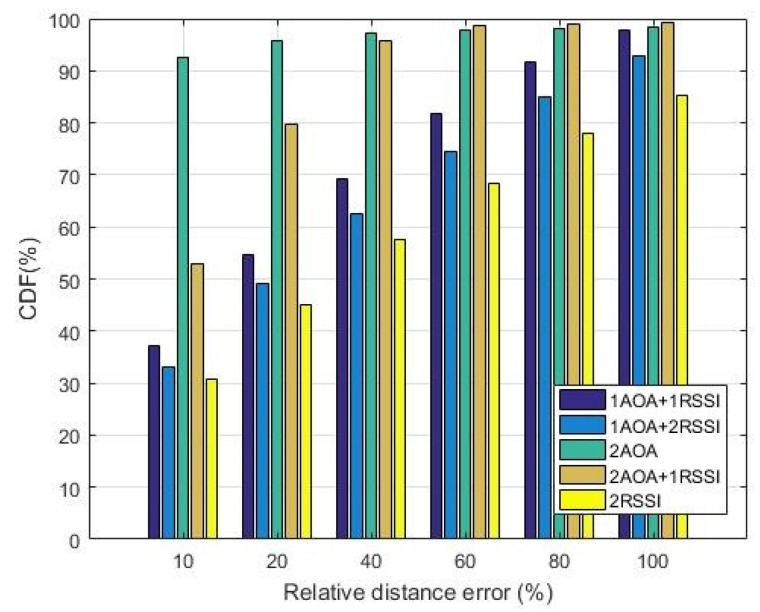
Accuracy comparison at SNR = 0 dB ($\delta^{2}=0$).

**Figure 14:**
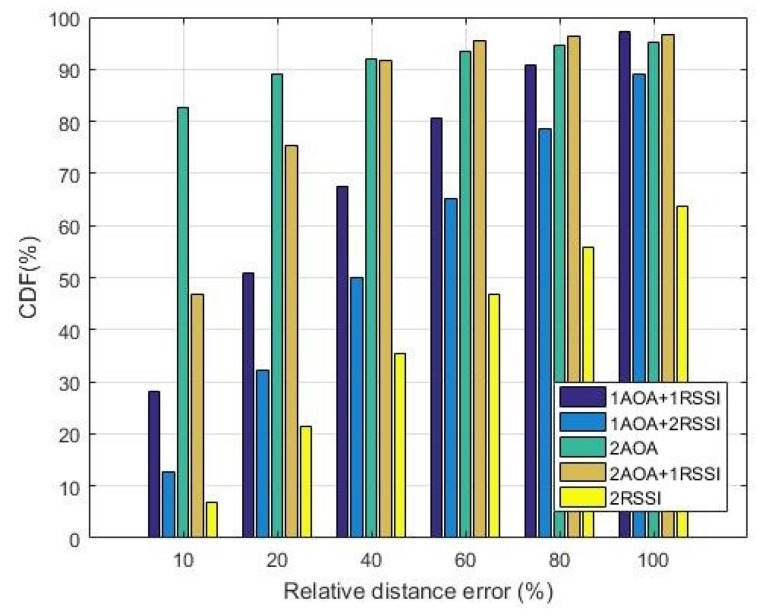
Accuracy comparison at SNR = 0 dB ($\delta^{2}=1$).

Figure 13 and Figure 14 show the accuracy performance of five methods using at most two anchors without and with shadowing at SNR = 0 dB. Cumulative distribution functions shown in these figures represent the probability of the nodes located within a certain RDE range. The instantaneous realizations of noise were considered since this model is more realistic for VANET at a low SNR, as explained in Section 5.1.2.

The figures show that the two most accurate localization methods are 2AOA and 2AOA + 1RSSI, which outperform all other methods, especially at the low RDE range, even for SNR = 0 dB. For instance, given the RDE of up to 10%, the probability of accurate localization of 2AOA is 93% in Figure 13 and 83% in Figure 14. In other words, if the distance from the true position of the unknown node to the anchor is 100 m, its estimated positions will have errors of up to 10 m 93% of the time in the case of no shadowing, and 83% of the time when shadowing with $\delta^{2}=1$ presents. A similar trend has also been observed for larger SNR values.

Figure 13 and Figure 14 also show that, from the accuracy perspective, the 2RSSI method consistently performs worst, compared to other methods, in a noisy environment regardless of having shadowing effects or not. This is the main weakness of the pure RSSI method.

It is also noticed that increasing the weighting of the AOA component and reducing that of the RSSI one will enhance the accuracy of the algorithms. For example, at RDE = 20%, the probability of locating nodes in 2AOA and 2AOA + 1RSSI is better than in 1AOA + 1RSSI by 43% and 31%, respectively. In a shadowing environment, the corresponding improvements are 42% and 32%. Another example is that 2AOA + 1RSSI better than 1AOA + 2RSSI by approximately 39% and 57% in Figure 13 and Figure 14, respectively. Recall from Figure 10 and Figure 11 that, at SNR = 0 dB, 2AOA and 2AOA + 1RSSI have a lower precision, compared to 1AOA + 1RSSI and 1AOA + 2RSSI. Therefore, at lower SNRs, the 2AOA and 2AOA + 1RSSI methods have a higher accuracy, but a lower precision, compared to the 1AOA + 1RSSI and 1AOA + 2RSSI methods. At high SNRs, however, both accuracy and precision of the 2AOA and 2AOA + 1RSSI methods are better than those in the 1AOA + 1RSSI and 1AOA + 2RSSI methods.

It can be deduced from the aforementioned analysis that good accuracy and good precision of the 2AOA and 2AOA + 1RSSI methods can both be achieved by increasing the SNR (i.e., transmitted power) when shadowing presents. Hence, automatic power control algorithms are highly recommended for these localization methods in shadowing environments. The transmitted power will be automatically increased when the channel experiences high shadowing and will be set back to normal when shadowing is negligible.

## 6. Conclusions

In this paper, we propose a mathematical model to examine shadowing effects for some common range-based methods, including RSSI, AOA and their weighted combinations. The paper then evaluates both precision and accuracy of numerous weighted combinations of RSSI and AOA methods under the effect of shadowing. As opposed to one’s intuition, the increase of the number of anchor nodes might not necessarily lead to the improvement of localization precision and accuracy. Our simulations also indicate that shadowing effects have a significant impact on both accuracy and precision of localization techniques, and that the RSSI component is more susceptible to shadowing than the AOA one. As a result, increasing the weight of the AOA component and reducing that of the RSSI one help improve both accuracy and precision at a high SNR range. Automatic power control algorithms can be used to increase the transmitted power when the channel experiences serious shadowing in order to achieve both good accuracy and good precision.

Besides shadowing effects, multipath propagation can also degrade the localization performance significantly in VANETs. Thus, our future work would be considering the effect of multipaths to the accuracy and precision of non-GPS localization. Our future work also includes the performance analysis of weighted combination algorithms in correlated fading channels [70,71] between antenna elements and the use of multi-antenna Orthogonal Frequency Division Multiplexing (OFDM) [72,73,74,75] techniques for non-GPS localization in multipath propagation environments.

## Figures and Tables

**Table 1:** Classification of Measuring Techniques.

Method	Bearing Measurement	Advantages	Disadvantages	Literature
Time of Arrival (TOA)	Distance	Simple to calculate	Require strict synchronization	[36,37,38,39,40]
Time of Arrival (TOA)	Distance	Simple to calculate	Require strict synchronization	[36,37,38,39,40]
Time Difference of Arrival (TDOA)	Distance	Asynchronous process	Time delay can be large and require large bandwidth	[41,42,43,44,45,46,47,48]
Frequency Difference of Arrival (FDOA) (i.e., Differential Doppler)	Distance	Robust for moving nodes	Hard to merely use FDOA to locate nodes because of its non-linear equation. FDOA is normally combined with TDOA	[43,44,49,50,51,52]
Received Signal Strength Indicator (RSSI)	Distance	Simplest method and do not require complicated hardware	Need preliminary knowledge on the propagation environment and subjective to noise	[12,13,14,15,23,53,54,55,56,57,58,59,60,61,62,63]
Angle of Arrival (AOA)	Angle	Robust to noise	More complex and expensive than other types	[3,4,5,16,26,28,29,64,65,66]
Power Difference of Arrival (PDOA)	Distance	Do not need many anchors in the network	Affected by shadowing and noise effects	[67,68]

**Table 2:** Summary of localization methods.

Number of Anchors	Method	Measurements	Mathematical Formulas	Graphical Representation
1	1AOA + 1RSSI	α, $d_{A}$	$$\begin{array}{ccc} x_{M} & = & x_{A}+d_{A}\sin\left(\alpha \right)\\ y_{M} & = & y_{A}+d_{A}\cos\left(\alpha \right) \end{array}$$	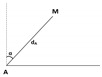
2	2AOA	α,β	$$\begin{array}{ccc} y_{M} & = & y_{A}+\frac{x_{B}-x_{A}}{\tan\left(\alpha \right)-\tan\left(\beta \right)}\\ x_{M} & = & x_{A}+\left(y_{M}-y_{A}\right)\tan\left(\alpha \right) \end{array}$$	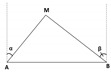
2	2RSSI	$d_{A},d_{B}$	$$\begin{array}{ccc} M & = & \left(A,d_{A}\right)\cap \left(B,d_{B}\right) \end{array}$$	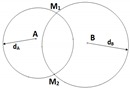
2	1AOA + 2RSSI	α, $d_{A}$, $d_{B}$	$$\begin{array}{ccc} M_{1} & = & \left(A,d_{A}\right)\cap \left(B,d_{B}\right)\\ x_{M_{2}} & = & x_{A}+d_{A}\sin\left(\alpha \right)\\ y_{M_{2}} & = & y_{A}+d_{A}\cos\left(\alpha \right)\\ M & = & (M_{1}+M_{2})/2 \end{array}$$	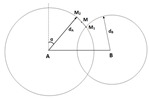
2	2AOA + 1RSSI	α, β, $d_{A}$	$$\begin{array}{ccc} x_{M_{1}} & = & x_{A}+d_{A}\sin\left(\alpha \right)\\ y_{M_{1}} & = & y_{A}+d_{A}\cos\left(\alpha \right)\\ y_{M_{2}} & = & y_{A}+\frac{x_{B}-x_{A}}{\tan\left(\alpha \right)-\tan\left(\beta \right)}\\ x_{M_{2}} & = & x_{A}+\left(y_{M}-y_{A}\right)\tan\left(\alpha \right)\\ M & = & (M_{1}+M_{2})/2 \end{array}$$	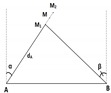
2	2AOA + 2RSSI	α,β, $d_{A},d_{B}$	$$\begin{array}{ccc} x_{M_{1}} & = & x_{A}+d_{A}\sin\left(\alpha \right)\\ y_{M_{1}} & = & y_{A}+d_{A}\cos\left(\alpha \right)\\ x_{M_{2}} & = & x_{A}+d_{B}\sin\left(\beta \right)\\ y_{M_{2}} & = & y_{A}+d_{B}\cos\left(\beta \right)\\ M & = & (M_{1}+M_{2})/2 \end{array}$$	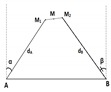
3	3RSSI	$d_{A},d_{B}$, $d_{C}$	$$\begin{array}{ccc} M_{1} & = & \left(A,d_{A}\right)\cap \left(B,d_{B}\right)\\ M_{2} & = & \left(A,d_{A}\right)\cap \left(C,d_{C}\right)\\ M_{3} & = & \left(B,d_{B}\right)\cap \left(C,d_{C}\right)\\ M & = & (M_{1}+M_{2}+M_{3})/3 \end{array}$$	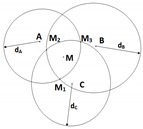
3	Weighted 3RSSI	$d_{A},d_{B}$, $d_{C}$	$$\begin{array}{ccc} M_{1} & = & \left(A,d_{A}\right)\cap \left(B,d_{B}\right)\\ M_{2} & = & \left(A,d_{A}\right)\cap \left(C,d_{C}\right)\\ M_{3} & = & \left(B,d_{B}\right)\cap \left(C,d_{C}\right)\\ M & = & \frac{\frac{M_{1}}{d_{B}+d_{C}}+\frac{M_{2}}{d_{A}+d_{C}}+\frac{M_{3}}{d_{A}+d_{B}}}{\frac{1}{d_{B}+d_{C}}+\frac{1}{d_{A}+d_{C}}+\frac{1}{d_{A}+d_{B}}} \end{array}$$	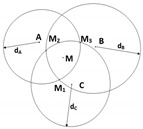

## Abbreviations

The following abbreviations are used in this manuscript:

AOA	Angle of Arrival
FDOA	Frequency Difference of Arrival
GPS	Global Positioning System
MSE	Mean Square Error
MUSIC	Multiple Signal Classification
OFDM	Orthogonal Frequency Division Multiplexing
PDOA	Power Difference of Arrival
RDE	Relative Distance Error
RF	Radio Frequency
RSSI	Receive Signal Strength Indicator
SNR	Signal to Noise Ratio
TDOA	Time Difference of Arrival
VANET	Vehicular Ad-Hoc Network
